# Coupling ultracold atoms to a superconducting coplanar waveguide resonator

**DOI:** 10.1038/s41467-017-02439-7

**Published:** 2017-12-21

**Authors:** H. Hattermann, D. Bothner, L. Y. Ley, B. Ferdinand, D. Wiedmaier, L. Sárkány, R. Kleiner, D. Koelle, J. Fortágh

**Affiliations:** 1 CQ Center for Quantum Science in LISA+, Physikalisches Institut, Eberhard Karls Universität Tübingen, Auf der Morgenstelle 14, D-72076 Tübingen, Germany; 2 Present Address: Kavli Institute of Nanoscience, Delft University of Technology, PO Box 5046, 2600 GA Delft, The Netherlands

## Abstract

Ensembles of trapped atoms interacting with on-chip microwave resonators are considered as promising systems for the realization of quantum memories, novel quantum gates, and interfaces between the microwave and optical regime. Here, we demonstrate coupling of magnetically trapped ultracold Rb ground-state atoms to a coherently driven superconducting coplanar resonator on an integrated atom chip. When the cavity is driven off-resonance from the atomic transition, the microwave field strength in the cavity can be measured through observation of the AC shift of the atomic hyperfine transition frequency. When driving the cavity in resonance with the atoms, we observe Rabi oscillations between hyperfine states, demonstrating coherent control of the atomic states through the cavity field. These observations enable the preparation of coherent atomic superposition states, which are required for the implementation of an atomic quantum memory.

## Abstract (web summary)

Using ultracold atoms in hybrid quantum devices is an interesting yet challenging task with possible applications for quantum storage. Here the authors demonstrate coherent magnetic coupling of an ensemble of ultracold rubidium atoms to a superconducting coplanar waveguide resonator.

## Introduction

Hybrid quantum systems of superconductors and atomic spin ensembles have been proposed^1–3^ for quantum information processing to overcome the limited coherence of superconducting qubits^4,5^. In the envisioned hybrid system, information is processed by fast superconducting circuits and stored in a cloud of cold atoms, which serves as a quantum memory^6–8^. Information is transferred between the two quantum systems using a superconducting coplanar waveguide resonator as a quantum bus. In recent years, coupling between superconducting structures and spin systems such as nitrogen vacancy centers^9–13^ and ions in solid-state systems^14,15^ has been observed. Cold atoms coupled to superconducting resonators would, furthermore, enable the implementation of novel quantum gates^16–19^, the realization of a microwave-to-optical transducer^20,21^, and on-chip micromasers^22^. The interaction between Rydberg atoms and three-dimensional superconducting microwave resonators has been a rich research topic, especially with regard to atom–photon interactions on the fundamental level^23^. Research on planar superconducting structures, however, holds the promise of switchable interactions between the subsystems, integration with scalable solid-state circuitry^24–26^, and long information storage times in the atomic ensemble. While long coherence times in cold atoms have been studied extensively^27–31^ and trapping and manipulation of atoms in the vicinity of superconducting chips has  been demonstrated in a series of experiments^32–37^, coupling between trapped atoms and planar superconducting resonators has not been shown yet.

In this article, we demonstrate magnetic coupling of ultracold magnetically trapped atoms to a superconducting coplanar waveguide resonator operated at temperatures around 6 K. The cavity is near resonant with the atomic hyperfine splitting of ^87^Rb and coherently driven by an external microwave synthesizer. We investigate both the dispersive and the resonant coupling regime. By driving the cavity off-resonantly with respect to the atoms, the atomic states reveal an AC-Zeeman shift under the influence of the microwave (MW) field^38^. This leads to a shift of the atomic transition frequency, which is measured by Ramsey interferometry. We use the AC-Zeeman shift to reconstruct the microwave intensity in the coplanar resonator. In contrast, when the cavity is driven at a frequency corresponding to an atomic transition, Rabi oscillations between atomic hyperfine states are observed.

Our measurements present a vital step toward the realization of an atom–superconductor hybrid system, paving the way toward the implementation of an atomic quantum memory coupled to a superconducting quantum circuit and the realization of microwave-to-optical transducers.

## Results

### Atomic ensembles trapped in a coplanar waveguide resonator

**Fig. 1:**
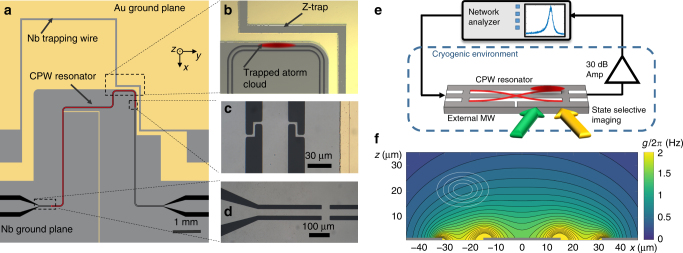
A superconducting atom chip for coupling ultracold atoms to a coplanar resonator. **a** Schematic top view of the superconducting atom chip, comprising a Z-shaped trapping wire and a coplanar microwave resonator (center conductor marked in red). Parts of the niobium ground planes have been replaced by gold to circumvent the Meissner effect and facilitate magnetic trapping. The slit in the lower ground plane prevents the formation of a closed superconducting loop. **b** Optical microscope image of the trapping region with the position of the atoms trapped close to the antinode of the resonator. During the measurements, trapping is purely provided by persistent supercurrents around the upper cavity gap and external fields. **c** Microscope image of the coupling inductances at the output of the resonator and **d** at the input of the resonator. **e** Scheme of the measurement setup. Atoms are coupled to a driven coplanar waveguide resonator and detected by state-selective absorption imaging. For Ramsey experiments in the dispersive regime, additional external microwave fields are used to manipulate the atoms. **f** Simulated coupling strength *g*/2*π* (Hz) between a single ground-state atom and a single photon in the cavity, resonant to the |1, −1〉 → |2, 0〉 transition. The white lines indicate positions of equal density for an atomic cloud of temperature *T*_at_ = 800 nK in the trap, corresponding to 20, 40, 60, and 80% of the density in the center

For our experiments, we magnetically trap an ensemble of ultracold ^87^Rb atoms in the state 5*S*_1/2_ *F* = 1, $m_F = - 1: = \left| {1, - 1} \right\rangle$ close to a coplanar microwave resonator on an integrated atom chip. The chip comprises two essential structures: i) a Z-shaped wire for magnetic trapping of neutral atoms and ii) a superconducting coplanar waveguide (CPW) resonator (Fig. 1a, b).

The CPW resonator is an inductively coupled half-wavelength cavity^39^ with a fundamental mode resonance frequency of *ω*_Res_ ≈ 2*π* · 6.84 GHz and a linewidth of *κ *≈ 2*π *· 3 MHz corresponding to a quality factor of *Q* ≈ 2200 in the temperature range (*T* = 6–7 K) relevant for the experiments described here. By varying the temperature of the atom chip, the resonance frequency of the microwave cavity can be tuned by about 30 MHz, where the atomic hyperfine transition frequency *ω*_HF_ = 2*π*  · 6.8347 GHz lies within this tuning range. Details on the chip design and fabrication methods can be found in Supplementary Note 1 of this article, and details on the cavity parameters and their temperature dependence in Supplementary Notes 2 and 3.

With the coupling inductors (Fig. 1c, d), the microwave cavity gap close to the Z trap provides a closed superconducting loop on the chip, in which the total magnetic flux is conserved. The other resonator gap does not form a closed loop, as the lower ground plane has been cut to avoid flux trapping. We take advantage of the flux conservation by freezing a well-defined amount of magnetic flux into the closed loop during the chip cooldown. A conservative magnetic trapping potential for the Rb atoms in the vicinity of the cavity mode is formed by the combination of flux-conserving loop currents and a homogeneous external field^31,40^. A homogeneous offset field along the *y*-axis *B*_off_ = 0.323 mT is additionally applied to ensure a nonzero magnetic field amplitude in the trap minimum to avoid spin-flip losses.

*N*_at_ ~ 10^5^ atoms are magnetically trapped at a distance of ~20 μm above one of the CPW gaps and close to one of the ends of the cavity, where the antinodes of the standing microwave magnetic fields are located, cf. Fig. 1b, e. At this position, the magnetic MW field of the transversal wave in the cavity is oriented perpendicular to the quantization axis of the atomic spins (*y* direction). Figure 1f depicts the coupling to the magnetic MW field of the cavity, obtained from finite-element simulations (Supplementary Note 4), in a cross-sectional view of the resonator. Solid white lines indicate the calculated positions of equal atomic density for an atomic cloud of 800 nK. From the MW field amplitude at the position of the atoms, we estimate an average single-atom single-photon coupling strength of $g = \vec \mu \cdot \vec B_{{\mathrm{ph}}} \approx 2\pi \cdot 0.5{\kern 1pt} {\mathrm{Hz}}$. The magnetic MW field and thus the coupling can be considered constant along the atomic cloud with an extension of ~100 μm in *y* direction, which is about two orders of magnitude smaller than the cavity and thus the wavelength. For the experiments described in this article, the cavity is driven by an external microwave synthesizer. In the limit of high photon numbers $n_{{\mathrm{ph}}} \gg N_{{\mathrm{at}}}$ explored in this article, the cavity field can be treated classically, and the collective coupling between an atom and the cavity is small compared to the damping rate. In the classical regime, the atoms couple individually to the cavity field, hence, the Rabi frequency is independent of the number of atoms in the cavity^41^.

### Sensing the cavity field with cold atoms

**Fig. 2:**
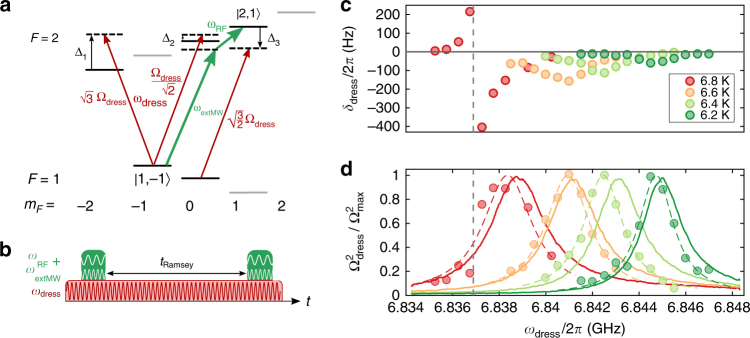
Probing the off-resonant cavity field with cold atoms. **a** Level scheme of the ^87^Rb ground-state manifold. The external MW and RF frequencies used for driving the two-photon transitions for the Ramsey scheme (green) and the off-resonant coupling of the cavity field to the relevant states are shown (red). **b** Experimental timing for the Ramsey sequence. The cavity field (red) is driven throughout the interferometric sequence. **c** Measured shift of the Ramsey frequency vs. frequency of the field in the superconducting microwave resonator for different chip temperatures. The sign change in the 6.8 K curve occurs at crossing the |1, 0〉 → |2, 1〉 transition, i.e., when Δ_3_ = 0, as indicated by dashed vertical line. **d** Data points: Calculated microwave intensity ${\mathrm{\Omega }}_{{\mathrm{dress}}}^2$ based on the measurements of *δ*_dress_. The colored dashed lines are Lorentzian fits to the data points. The solid lines are the measured transmission spectra of the microwave resonator

When driving the resonator at a frequency *ω*_dress_ off-resonant to the atoms, the atomic transition is shifted by the MW field. This AC-Zeeman shift can be experimentally detected and used to reconstruct the intensity of the cavity field. We measure the frequency of the atomic transition between the magnetically trapped states |1, −1〉 and |2, 1〉 using time-domain Ramsey interferometry. The two states exhibit the same first-order Zeeman shift, thereby strongly reducing the sensitivity of the transition frequency to magnetic fields. For the Ramsey measurements, the atoms are prepared in a coherent superposition driven by a pulsed MW field *ω*_extMW_ from an external antenna and an additional radio frequency of *ω*_RF_ fed to the Z-shaped trapping wire (green arrows in Fig. 2a). After a variable time *T*_Ramsey_, a second MW + RF pulse is applied and the relative population in the two states is measured. The populations in the two states oscillate with the difference between the atomic frequency and the external frequency, *ω*_at_ − (*ω*_extMW_ + *ω*_RF_). During the Ramsey sequence, the CPW cavity is driven by a field with a variable angular frequency *ω*_dress_ that is off-resonant to the atomic transition (Fig. 2b). This leads to an AC shift of the levels which depends on the detuning Δ between *ω*_dress_ and the atomic transition frequency. For a simple two-level system, the off-resonant field shifts the atomic states by $\delta _{{\mathrm{dress}}} = \pm \frac{{{\mathrm{\Omega }}_{{\mathrm{dress}}}^2}}{{\mathrm{\Delta }}}$, where Ω_dress_ denotes the Rabi frequency of the dressing field and Δ = *ω*_dress_ − *ω*_0_ is the detuning between the dressing field and the atomic transition frequency. The plus (minus) sign is valid for the ground (excited) state. The level scheme of the atoms involving all relevant fields is depicted in Fig. 2a. For a MW field which is linearly polarized perpendicular to the quantization axis, as it is in our case, the cavity field induces *σ* ^−^ and *σ* ^+^-transitions with equal field strength, as depicted by the red arrows. This field hence couples the state |1, −1〉 to the states |2, −2〉 and |2, 0〉. The state |2, 1〉, on the other hand, is coupled to state |1, 0〉. This leads to a shift in the two-photon transition frequency |1, −1〉 → |2, 1〉 by

1 $$\delta _{{\mathrm{dress}}} = - {\mathrm{\Omega }}_{{\mathrm{dress}}}^2 \cdot \left( {\frac{3}{{{\mathrm{\Delta }}_1}} + \frac{{1{\mathrm{/}}2}}{{{\mathrm{\Delta }}_2}} + \frac{{3{\mathrm{/}}2}}{{{\mathrm{\Delta }}_3}}} \right),$$

which is measured in our experiment (Supplementary Note 5 for details). Here, Δ_*i*_,*i* ∈ {1, 2, 3} denotes the detuning to the relevant atomic hyperfine transition. The numerical factors in the numerator are determined by the Clebsch–Gordan coefficients of the transitions.

For the measurement, the power of the microwave fed to the resonator and the magnetic offset field (*B*_off_ = 0.315 ± 0.003 mT) is held constant.

The measured frequency shift *δ*_dress_ in the Ramsey experiment is shown in Fig. 2c. As visible in the curve measured at *T* = 6.8 K, the dressing shift changes sign when the frequency of the dressing field is crossing an atomic resonance. Variation of the dressing frequency affects the shift in two ways, via the detuning to the atomic transitions and via a change in the microwave intensity in the resonator. Knowing the detuning to all involved levels, the normalized power of the microwave in the resonator, which is proportional to the square of the resonant Rabi frequency ${{\Omega }}_{{\mathrm{dress}}}^2$, can be deduced from the dressing shift. The calculated Rabi frequencies Ω_dress_ according to Eq. (1) are shown as circles in Fig. 2d. The measurement was repeated for different temperatures of the superconducting chip, corresponding to different cavity resonance frequencies. The result is compared with transmission spectra measured using a programmable network analyzer (solid lines in Fig. 2d). All curves are normalized to their maxima for the sake of comparability. Lorentzian curves (dashed lines) fitted to the data points match the transmission spectra closely in center frequency and peak width, which is on the order of *κ*/2*π* ≈ 2 − 3 MHz. We note that there is a slight systematic offset between the reconstructed Rabi frequencies and the transmission curves. Possible explanations are trapping of Abrikosov vortices during the Ramsey measurement due to cycling of applied magnetic fields, which are known to shift the resonator frequency to lower values, or a small temperature difference (<50 mK) between the two measurements.

### Coherent control of atomic states with cavity fields

**Fig. 3:**
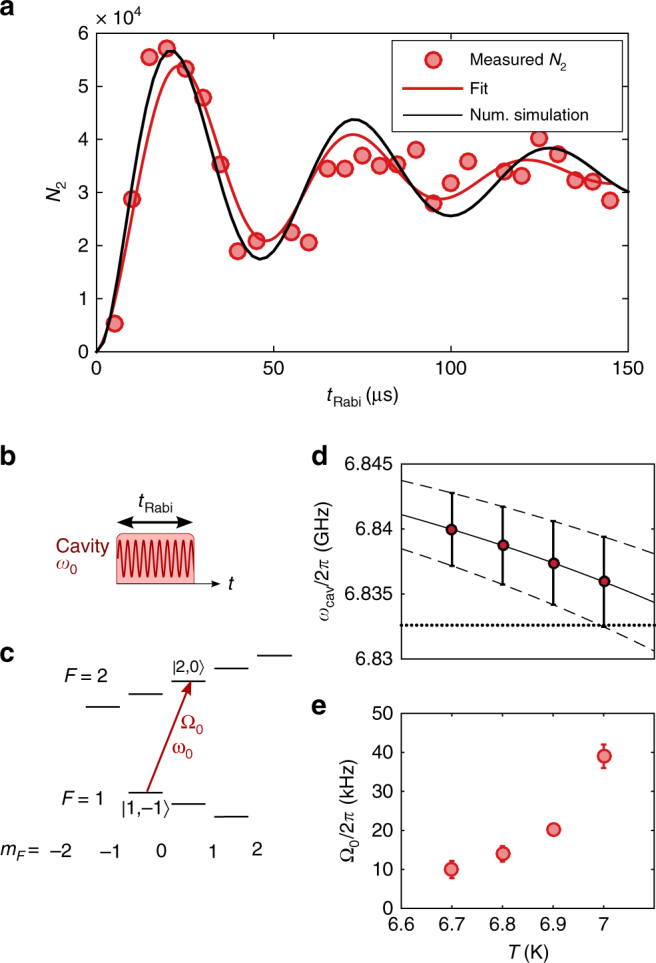
Cavity-driven Rabi oscillations. **a** Measurement of the atoms in state |2, 0〉 reveals resonant Rabi oscillations between |1, −1〉 and |2, 0〉 for a cavity-driving frequency of *ω*_0_ = 2*π* · 6.83242 GHz. The chip temperature was set to *T* = 6.9 K. The red solid line is a fit to the damped oscillation, and the black line shows the result of the numerical simulations. **b** Timing sequence and **c** level scheme for the driven one-photon Rabi oscillations. **d** Temperature dependence of the cavity resonance frequency. The circles and error bars indicate the peak and the width (±*κ*) of the cavity line obtained from fits to the resonator transmission data. The solid and dashed lines indicate the fitted temperature dependence of the cavity frequency and linewidth (Supplementary Note 3 for details). The horizontal dotted line indicates the driving frequency, corresponding to the atomic resonance. **e** Temperature dependence of the Rabi frequency. While the cavity is driven at the same frequency *ω*_0_ for all measurements, the temperature dependence of the cavity resonance leads to a change in the microwave intensity. Error bars indicate the confidence interval of the Rabi frequency measurement

When the electromagnetic cavity field is resonant with one of the (allowed) atomic transitions, the atoms undergo coherent Rabi oscillations between the ground and excited state (Fig. 3a). The observation of these oscillations demonstrates coherent control over the internal atomic degrees of freedom. The Rabi frequency is given by ${\mathrm{\Omega }}_0 = {\vec{\mathrm \mu}} \cdot \vec B_{{\mathrm{MW}}}$, where $\vec \mu$ is the atomic magnetic moment and $\vec B_{{\mathrm{MW}}}$ is the amplitude of the oscillating magnetic MW field. For the observation of these oscillations, we drive the cavity with a frequency of *ω*_0_ = 2*π* · 6.83242 GHz, which is in resonance with the atomic transition |1, −1〉 → |2, 0〉, but detuned roughly by twice the cavity linewidth *κ* from the cavity resonance (*ω*_cav_ ≈ 2*π* · 6.839 GHz) at a chip temperature *T* = 6.9 K (Fig. 3b, c). By state-selective absorption imaging of the atoms, we observe resonant Rabi oscillations between the states |1, −1〉 and |2, 0〉 with a Rabi frequency Ω_0_ ≈ 2*π* · 20 kHz. By variation of the chip temperature between *T* = 6.7 and 7.0 K, the cavity frequency is shifted with respect to the atomic transition (Fig. 3d). This leads to a measurable change in the resonant Rabi frequency due to the altered MW power in the cavity, as visible in Fig. 3e. Here, the Rabi frequency increases with higher temperatures, as the cavity frequency approaches the atomic transition frequency. For temperatures around *T* = 7.2 K, the cavity resonance is shifted to coincide with the atomic resonance. However, at this temperature, the critical current of the superconducting coupling inductances is too low to support a stable magnetic trap.

We observe a damping in the single-photon Rabi oscillations with a time constant of *τ* ≈ 50 µs. This damping is a result of the dephasing due to the inhomogeneous MW field of the cavity and the fact that Rabi oscillations are driven between two states with different magnetic moments. The magnetically trapped state |1, −1〉 is subjected to an energy shift of ~2*πħ* · 7 MHz/mT, while the untrapped state |2, 0〉 is in first order insensitive to magnetic fields. As a consequence, the resonance frequency between the two states is not uniform across the cloud, and the atoms are only exactly on resonance at the center of the trap. A numerical simulation of a thermal cloud at *T*_at_ = 2 µK trapped in a harmonic magnetic potential 20 µm above the cavity gap shows a damping time in excellent agreement with our measurement. We can estimate the number of photons in the resonator using the measured Rabi frequency and the simulated coupling strength per photon. Assuming a Rabi frequency of 20 kHz, we estimate the number of photons in the cavity *n*_Ph_ ≈ 1.6 × 10^9^, so that the thermal occupation of the cavity (*n*_th_ ~ 20) is negligible.

**Fig. 4:**
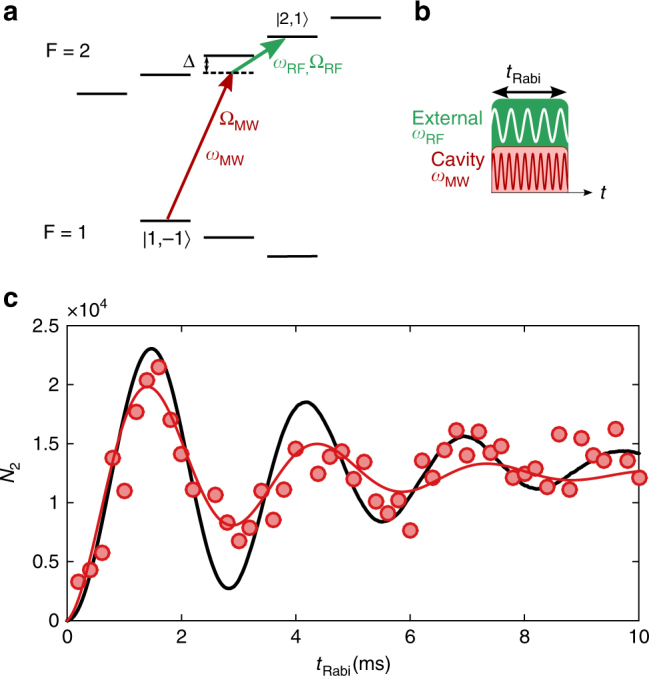
Two-photon Rabi oscillations. **a** Level scheme and **b** timing sequence for the two-photon Rabi oscillations between the trapped states |1, −1〉 and |2, 1〉. **c** Observation of two-photon Rabi oscillations between states |1, −1〉 and |2, 1〉 (circles), and fit of the damped oscillation (red), yielding a damping time of *τ* = 5 ms due to the inhomogeneity of the MW field amplitude across the cloud. The black solid line shows a numerical simulation of the state evolution for an ensemble of thermal atoms moving in the trap

In order to exploit the long coherence times of cold atoms, it is necessary to create superpositions between appropriate atomic states, which can both be trapped in the cavity. For ^87^Rb, such a state combination consists of the hyperfine levels |1, −1〉 and |2, 1〉, which can both be trapped magnetically and exhibit excellent coherence properties. To this end, we start with an atomic cloud at a lower temperature of *T*_at_ = 800 nK and *N*_at_ ~ 3 × 10^4^ atoms in the state |1, −1〉. In order to prepare a coherent superposition of the two states, we drive the cavity with the MW field *ω*_MW_ and employ an additional external RF field *ω*_RF_, with a detuning of Δ = 2*π* · 300 kHz to the intermediate state |2, 0〉 (cf. Fig. 4a). If the two corresponding Rabi frequencies are small compared to the intermediate detuning, i.e., ${\mathrm{\Omega }}_{{\mathrm{MW}}}$, ${\mathrm{\Omega }}_{{\mathrm{RF}}} \ll {\mathrm{\Delta }}$, the population of the intermediate state can be neglected. In this case, the two-photon Rabi frequency Ω_2Ph_ can be calculated by adiabatic elimination of the intermediate state Ω_2Ph_ = Ω_MW_Ω_RF_/2Δ^42^. By driving the two fields with variable pulse lengths (Fig. 4b), we observe Rabi oscillations with Ω_2Ph_ = 2*π* · 340 Hz, and a dephasing on the order of *τ *~ 5 ms (Fig. 4c). A numerical simulation of an ensemble of noninteracting atoms in a magnetic trap reveals damping on the same timescale. As in the one-photon case, the dephasing is mainly due to the variation of the microwave field strength over the size of the atomic cloud (Supplementary Note 6).

## Discussion

To make the presented cold atom–superconductor hybrid device a useful high-coherence quantum resource, several aspects need to be addressed and optimized. In particular, dephasing during the Rabi pulses should be reduced and the coupling between atoms and the cavity increased.

Dephasing due to inhomogeneous coupling, as seen in the experiment above, can be a limitation for the high-fidelity creation of superposition states needed in information processing. The inhomogeneity seen by the atomic ensemble can be reduced by reducing the cloud temperature, yielding smaller cloud extension in the trap (Supplementary Note 6). Several experiments have furthermore shown that reliable superpositions or quantum gates can be achieved in spite of this temporal or spatial variation of Rabi frequencies, as the related dephasing can be overcome using more elaborate MW and RF pulses using optimal control theory^43,44^.

Furthermore, future experiments should be performed at chip temperatures in the mK regime to reduce the number of thermal photons, so, different ways to tune the cavity frequency need to be employed. Tuning the cavity could be achieved by various means, such as using nonlinear kinetic inductances^45^, SQUIDs^46,47^, or mechanical elements, as demonstrated in ref. ^48^.

For our geometry, we have estimated the coupling between a single atom and a single cavity photon to be *g* ≈ 2*π* · 0.5 Hz. Various means can be used to increase the coupling strength between the atoms and the cavity field. By decreasing the width of the gap *W* between the center conductor and ground planes of the cavity, the magnetic field per photon could be increased according to *B*_ph_ ∝ 1/*W* ^2^, but would require the atoms to be trapped closer to the chip surface. By changing the resonator layout from CPW to lumped element resonator, the inductance and dimensions of the resonator could be decreased, leading to a significant enhancement of the current per photon and hence magnetic field *B*_ph_. Finally, the electric field of the cavity mode could be used to couple neighboring Rydberg states, exploiting the large electric dipole moments of Rydberg states^49^. A similar experiment has been demonstrated with flying Rydberg atoms above a CPW transmission line^50^. For our geometry, the transition between the states 57*S*_1/2_ and 57*P*_3/2_ lies close to the third harmonic of our resonator. The dipole matrix element of this transition is *d *~ 2700* ea*_0_, yielding a single-photon single-atom coupling strength of *g*/2*π* ≈ 0.1 MHz.

In summary, we have experimentally demonstrated coupling of ultracold ground-state atoms to a driven superconducting CPW resonator. Coupling was shown both in resonant Rabi oscillation and in dressing the frequency of an atomic clock state pair. Future measurements will explore collective effects of cold atoms to the cavity mode and work toward strong coupling between the superconducting resonator and Rydberg atoms. These experiments are the first step toward the implementation of cold atoms as a quantum resource in a hybrid quantum architecture.

## Methods

### Atomic cloud preparation

The atomic ensemble is prepared in a room-temperature setup and transported to a position below the superconducting atom chip using an optical dipole trap that is moved using a lens mounted on an air-bearing translation stage (cf. ref. ^51^ for details). Atoms are subsequently trapped in a magnetic trap generated by currents in the Z-shaped Nb wire and an external homogeneous bias field. The Z-wire configuration leads to a Ioffe-Pritchard- type magnetic microtrap with a nonzero offset field *B*_off_ at the trap minimum. We load ~10^6^ atoms at a temperature of ~1 µK into the magnetic chip trap. After adiabatic compression, the cloud is transferred into the mode volume of the resonator by rotating the external bias field and switching off the current in the Z trap. Screening currents in the resonator, which conserve the flux in the closed superconducting loop, lead to the formation of a magnetic trap with oscillation frequencies *ω*_*x*_ = 2*π* · 400 s^−1^, *ω*_*y*_ = 2*π* · 25 s^−1^, and *ω*_*z*_ = 2*π* · 600 s^−1^ below the gap of the waveguide cavity, 20 µm from the chip surface. During the transfer into the tight trap, the atomic cloud is heated up to a temperature of *T*_at_ ~ 2 μK. At the cavity position, we perform radiofrequency evaporation to further cool the atomic ensemble.

### Experimental cycle and state-selective detection

In order to measure the atomic state, the following experimental cycle is repeated every ~26 s. After preparation of an atomic cloud, transporting it to the superconducting chip, and loading into the cavity, as described above, all atoms are in the hyperfine state |1, −1〉. Subsequently, we apply one MW (+RF) pulse of variable length *t*_Rabi_ for the measurement of Rabi oscillations, or two *π*/2-pulses of fixed length with a variable hold time *t*_Ramsey_ in-between for the Ramsey interferometry sequence. At the end of the sequence, we can measure the number of atoms in both of the states. First, the number of atoms in *F* = 2 is measured by illuminating the cloud with light resonant to the 5*S*_1/2_, *F* = 2 → 5*P*_3/2_, and *F* = 3 transition. The shadow of the atoms is imaged on a CCD camera and the measured optical density is used to determine the atom number. We then pump the atoms from *F* = 1 into *F* = 2 by illumination with a laser resonant with the 5*S*_1/2_, *F* = 1 → 5*P*_3/2_, and *F* = 2 transition. From the 5*P*_3/2_, *F* = 2 state, the atoms decay into 5*P*_1/2_, *F* = 2 in ~30 ns, and the atoms are imaged on a second CCD camera as described above.

### Data availability

The data that support the findings of this article are available from the authors on reasonable request.

## Electronic supplementary material

Supplementary Information
