# Supplementary material for: Coupling ultracold atoms to a superconducting coplanar waveguide resonator
Source: Nat Commun. 2017 Dec 21;8:2254. doi: 10.1038/s41467-017-02439-7 (PMC5740063; doi:10.1038/s41467-017-02439-7)
Supplement: Supplementary file 1 — Supplementary Information [file 41467_2017_2439_MOESM1_ESM.pdf]

SUPPLEMENTARY NOTE 1. ATOM CHIP DESIGN AND FABRICATION

Our atom chip combines two structures, a $Y = 100\,\mu$m wide Z-shaped superconducting Nb strip for the application of directed and low frequency currents as well as a superconducting coplanar waveguide resonator with a resonance frequency of $\omega_{\rm Res} \approx 2\pi \cdot 6.85\,$GHz, near-resonant with the ground state hyperfine transition frequency of ^{87}Rb atoms. All structures are patterned onto a $h_{\rm S} = 330\,\mu$m thick sapphire substrate by means of optical lithography, thin film deposition and microfabrication . A schematic of the atom trapping region on the chip is shown in Supplementary Figure 1a and a cross-sectional view along the dotted line in 1a is shown in 1b. The full chip layout is shown in Fig. 1a of the main paper.

The coplanar microwave resonator has a centre conductor width of $S = 30\,\mu$m and two ground planes, which are separated from the centre conductor by a gap of $W = 16\,\mu$m, targeting a characteristic impedance $Z_0 = 50\,\Omega$. In order to facilitate the magnetic trapping of atoms closely above the gaps of the waveguide structure, the magnetic field distorting superconducting ground planes had to be removed partially. As we observe strong parasitic resonances when parts of the ground planes are missing (probably due to a parasitic mutual inductance between the trapping wire and the waveguide structure and due to the excitation of chip resonances), we substituted the removed superconducting parts by a normal-conducting Au metallization layer, restoring a good ground connection along the whole resonator. Thus, the trapping wire is embedded into one of the ground planes and galvanically connected to all metallization parts on the chip. As superconductor we use niobium, and as normal conductor we use gold on top of a thin adhesion layer of titanium. The thicknesses of the three films are $h_{\rm Nb} = 500\,$nm, $h_{\rm Au} = 400\,$nm, and $h_{\rm Ti} = 4\,$nm, cf. Supplementary Figure 1b. Between the superconducting parts and the normal-conducting parts, there is a $O = 10\,\mu$m wide overlap region, ensuring a low contact resistance.

In order to minimize additional microwave losses induced by the presence of the normal conductor, we only replaced the superconductor by gold in the trapping region ($\sim 15\%$ of the total resonator length) and kept also a $G = 50\,\mu$m part of the ground plane in this region superconducting. The normal conducting region in between this remaining superconducting part of the ground plane and the superconducting trapping wire is $D + 2O = 120\,\mu$m wide, cf. Supplementary Figure 1b.

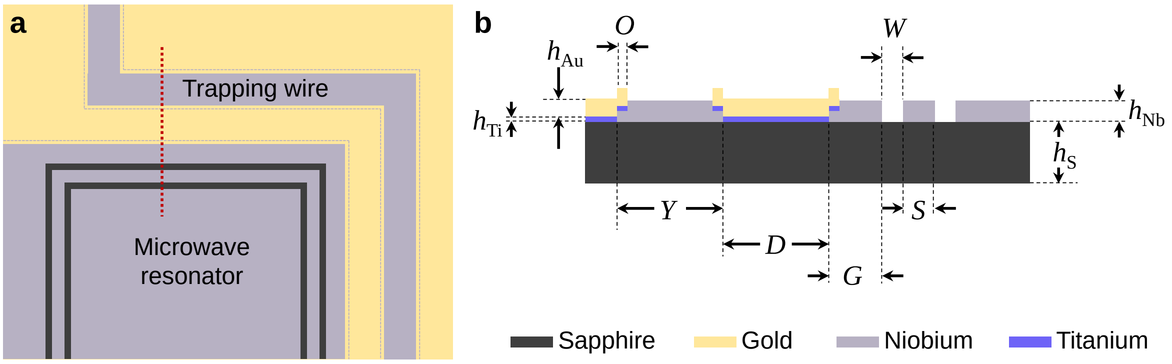

Supplementary Figure 1: **Atom chip layout and parameters. a** Schematic top view of the trapping region of the atom chip. A Z-shaped atom trapping wire passes by a coplanar microwave resonator structure. The trapping wire and the core region of the microwave resonator consist of superconducting Nb, the two structures are galvanically connected by a normal conducting gold layer in order to guarantee well-defined microwave properties. **b** Cross section along the red dotted line in 1a, depicting and defining all relevant materials, thicknesses and geometrical parameters of the device. Resonator centre conductor width $S = 30\,\mu$m, gap size $W = 16\,\mu$m, width of the superconducting ground plane $G = 50\,\mu$m. The Z-wire is $Y = 100\,\mu$m wide, the normal conducting region between resonator and Z-wire is $D = 100\,\mu$m wide. The thicknesses of the three films are $h_{\rm Nb} = 500\,$nm, $h_{\rm Au} = 400\,$nm, and $h_{\rm Ti} = 4\,$nm. Between the superconducting parts and the normal-conducting parts, there is a $O = 10\,\mu$m wide overlap. The chip substrate is $h_{\rm S} = 330\,\mu$m thick. Thicknesses are not to scale.

The device fabrication is schematically shown in Supplementary Figure 2. It starts with the DC magnetron sputtering of the Nb onto a bare r-cut Sapphire substrate. By means of optical lithography and SF_6 reactive ion etching, we pattern the superconducting parts. Next, we cover most of the superconducting parts – except for the $10\,\mu$m wide overlap region – with photoresist and deposit the normal conducting metal on top. To do so, we first remove 200 nm of the Nb in the overlap region by another SF_6 reactive ion etching step in order to get rid of photoresist residues and a possible native oxide layer on top of the Nb and in addition to reduce the substrate-Nb step height. Then, we in-situ deposited the Ti adhesion layer by means of electron beam evaporation and the Au layer by DC magnetron sputtering. We finalized the fabrication by lifting off the normal conducting parts in hot acetone supported by ultrasound.

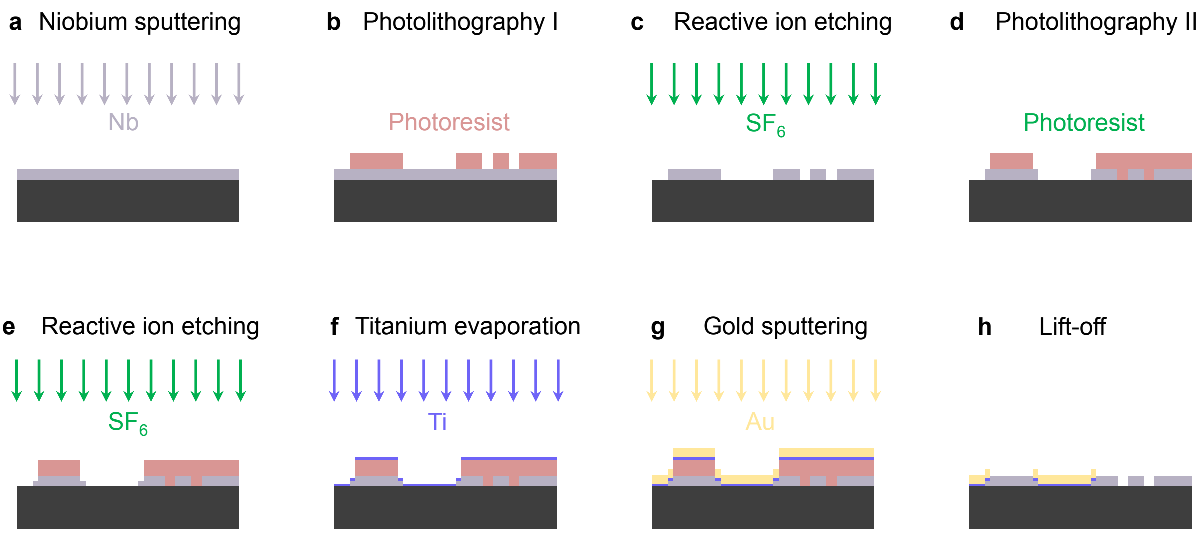

Supplementary Figure 2: **Atom chip fabrication**. Schematic fabrication sequence of the chip used in this experiment. Thicknesses are not to scale. **a** DC magnetron sputtering of Nb onto a Sapphire substrate. **b** Photolithopgraphy and **c** reactive ion etching defining the superconducting chip parts. **d** Protection of the superconducting parts with photoresist, except for a $O = 10\,\mu$m wide overlap edge region. **e** Removal of the native oxide in the overlap region and reduction of the substrate-Nb step height by reactive ion etching. **f** Electron beam evaporation of a titanium adhesion layer. **g** DC magnetron sputtering of Au. Steps **e-g** are performed in-situ. **h** Ultrasound assisted lift-off of Au/Ti in warm acetone.

SUPPLEMENTARY NOTE 2. CAVITY PARAMETERS

The microwave resonator used in this experiment is a half wavelength ($\lambda/2$) transmission line cavity based on a coplanar waveguide with charactersitic impedance $Z_0 \approx 50\,\Omega$ and attenuation constant α. The transmission line cavity has a length $l_0 \approx 9.3\,$mm and a fundamental mode resonance frequency $\omega_{\rm Res} = 2\pi \cdot 6.85\,$GHz at a temperature of $\sim 5\,$K. Around its resonance frequency, the waveguide resonator can be modelled as an inductively coupled series RLC circuit, cf. Supplementary Figure 3a and 3b with the equivalent lumped element resistance R, inductance L and capacity R [1]:

$$R = Z_0 \alpha l_0, \qquad L = \frac{\pi Z_0}{2\tilde{\omega}_{\rm Res}}, \qquad C = \frac{2}{\pi \tilde{\omega}_{\rm Res} Z_0} \tag{1}$$

where α is the attenuation constant of the coplanar waveguide and $\tilde{\omega}_{\rm Res}$ is the "uncoupled" resonance frequency, i.e., the resonance frequency corresponding only to the electrical length of the cavity.

For driving the resonator and reading out its frequency dependent response, the cavity is weakly coupled to two feedlines by shunt inductors between the centre conductor and the ground planes at both ends, cf. Fig. 1 of the main paper. The shunt inductors at the input port are shown in Supplementary Figure 3c. Each of the two superconducting shunts to ground is $36\,\mu$m wide and $16\,\mu$m long. With the software package 3D-MLSI [2], we determined each of the two shunt inductances to be $L_1 = 2.94\,$pH, giving a total input port coupling inductance $L_{\rm in} = L_1/2 = 1.47\,$pH.

At the output port, cf. Supplementary Figure 3e, the shunt inductors are $4\,\mu$m wide and $30\,\mu$m long, giving an inductance per shunt of $L_2 = 12.88\,$pH. Thus, the total inductance at the output port is $L_{\rm out} = L_2/2 = 6.44\,$pH.

For $\tilde{\omega}_{\rm Res} L_{\rm in}, \tilde{\omega}_{\rm Res} L_{\rm out} \ll Z_0$ the resonance frequency of the coupled circuit is shifted due to the coupling inductors according to

$$\omega_{\rm Res} = \frac{1}{\sqrt{(L + L_{\rm in} + L_{\rm out})C}}. \tag{2}$$

The external linewidth of the resonator due to losses through the input port is given by [1]

$$\kappa_{\rm ex1} = \omega_{\rm Res} \frac{\pi}{2} \frac{L_{\rm in}^2}{L^2} \approx 2\pi \cdot 7\,\text{kHz}. \tag{3}$$

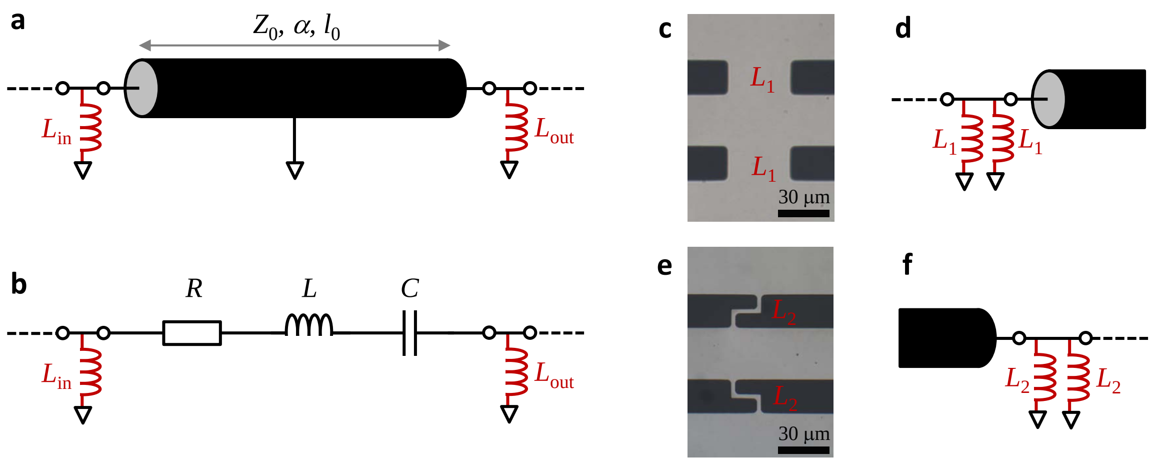

Supplementary Figure 3: **Resonator parameters and description**. **a** Schematic of an inductively coupled transmission line cavity as used in this experiment. The transmission line resonator is characterized by its characteristic impedance Z_0, its length l_0 and its attenuation constant α. The cavity is coupled at both ends to transmission feedlines via shunt inductors L_{in} and L_{out}. **b** Lumped element circuit equivalent of 3a with the equivalent resistor R, the equivalent inductor L and the equivalent capacitor C. **c** [**e**] shows an optical image of the input [output] coupling inductors of our device and **d** [**f**] shows its circuits equivalent. As in the coplanar waveguide geometry we have two parallel shunt inductors L_1 [L_2] to ground at the input [output] port, the total input [output] coupling inductance is given by $L_{\text{in}} = L_1/2$ [$L_{\text{out}} = L_2/2$].

For the output port, we find

$$\kappa_{\text{ex2}} = \omega_{\text{Res}} \frac{\pi}{2} \frac{L_{\text{out}}^2}{L^2} \approx 2\pi \cdot 134\,\text{kHz}. \tag{4}$$

These linewidths correspond to a total external linewidth

$$\kappa_{\text{ex}} = 2\pi \cdot 141\,\text{kHz} \tag{5}$$

or a total external quality factor

$$Q_{\text{ex}} = \frac{\omega_{\text{Res}}}{\kappa_{\text{ex}}} \approx 5 \cdot 10^4. \tag{6}$$

In liquid helium, at temperature $T_s = 4.2\,\text{K}$, we measure a total quality factor of $Q \approx 10000$, indicating that the majority of the losses is due to thermal quasiparticles in the superconductor as well as due to dissipation in the normal conducting parts and the interfaces between the different metals.

SUPPLEMENTARY NOTE 3. CAVITY TEMPERATURE DEPENDENCE

A. Temperature calibration

The magnetic penetration depth λ_{L} in a BCS superconductor shows a temperature dependence, which can be approximately captured by [3]

$$\lambda_{\text{L}}(T) = \frac{\lambda_{\text{L}}(T=0)}{\sqrt{1 - \left(\frac{T_s}{T_c}\right)^4}} \tag{7}$$

with the sample temperature T_s and the superconducting transition temperature T_c. The origin of this temperature dependence is the temperature dependence of the superconducting charge carrier density.

The total inductance of a superconducting resonator is given by the sum of the temperature independent geometric inductance L_g and the kinetic inductance, $L_k(T)$, which takes into account the kinetic energy of the superconducting charge carriers. For superconductors with a thickness larger than twice the penetration depth, the kinetic inductance is related to the magnetic penetration depth via

$$L_k(T) = \chi_g \mu_0 \lambda_{\mathrm{L}}(T), \tag{8}$$

where χ_g is a geometrical factor, taking into account the spatial distribution of the superconducting current density. In our samples, we have $h_{\mathrm{Nb}} = 500\,\mathrm{nm}$ and typically $\lambda_{\mathrm{L}}(T=0) \sim 100\,\mathrm{nm}$. Thus, up to $T_s/T_c \approx 0.95$, which is much larger than all values of T_s/T_c in our experiment, $h_{\mathrm{Nb}} > 2\lambda_{\mathrm{T}}$ is fulfilled.

In general, also the coupling inductors have a kinetic contribution, but due to $L \gg L_{\mathrm{in}}, L_{\mathrm{out}}$ in our device, we neglect this small correction here. With the temperature dependent kinetic inductance, the resonance frequency is given by

$$\omega_{\mathrm{Res}}(T) = \frac{\omega_{\mathrm{Res0}}}{\sqrt{1 + \frac{L_k(T)}{L_0}}}, \tag{9}$$

where $L_0 = L_g + L_{\mathrm{in}} + L_{\mathrm{out}}$ is the inductance of the cavity without the kinetic contribution and $\omega_{\mathrm{Res0}} = 1/\sqrt{L_0 C}$ is the resonance frequency for $L_k = 0$ (not for $T = 0$).

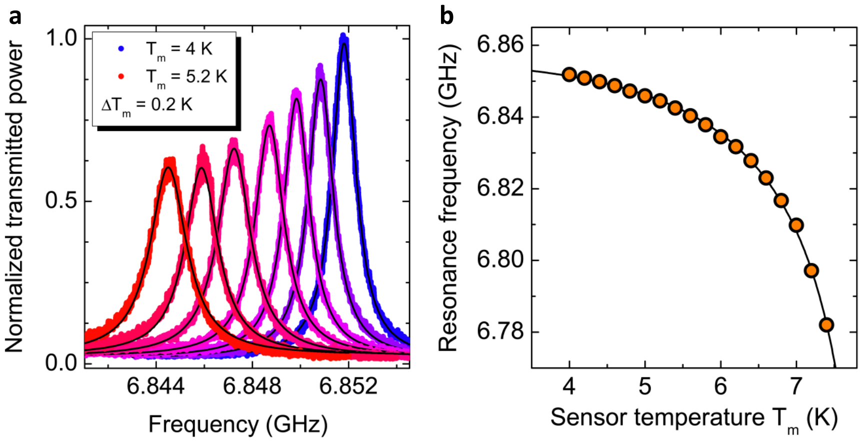

Supplementary Figure 4: **Temperature calibration**. **a** Cavity transmission spectra measured for sensor temperatures $4\,\mathrm{K} \leq T_m \leq 5.2\,\mathrm{K}$ in steps of $\Delta T_m = 0.2\,\mathrm{K}$. With increasing temperature, the resonance frequency shifts to lower values. Black lines are Lorentzian fits. **b** Cavity resonance frequency $\omega_{\mathrm{Res}}/2\pi$ vs sensor temperature. Circles are data extracted from the measurements and the black line is an analytical approximation curve (for details see text).

In our experiment, we take advantage of the temperature dependence of the cavity resonance frequency to tune it close to the atomic transition frequency. Figure 4a shows (smoothed) transmission spectra for different temperatures measured with the sensor mounted to the helium flow cryostat, which also hosts the chip. We observe the resonance frequency shifting towards lower values with increasing temperature. In Supplementary Figure 4b, we plot the extracted resonance frequency vs the measured temperature T_m.

As the thermometer is positioned inside the coldfinger of the flow cryostat $\sim 10\,\mathrm{cm}$ from the chip itself, we expect the sample temperature T_s to be different from the sensor temperature T_m by an offset temperature T_{off}, i.e.,

$$T_s = T_m + T_{\mathrm{off}}. \tag{10}$$

We note that we use a calibrated sensor and thus that the offset is not related to uncertainty of the sensor measurement, but due to the nature of the setup [4]. The chip and the microwave amplifier are mounted on a 10 cm high sample holder of oxygen-free high-conductivity copper. The cooling power of the chip is mainly limited by the thermal conductivity through the interfaces between the cryostat and the chip holder and between chip holder and the sapphire chip. Due to the requirement to have optical access to the chip region, 5 mm high slits have been cut into the thermal shield at 20 K, which encloses the coldfinger tip and the sample holder in order to minimize the thermal

radiation from the room temperature environment. The final temperature of the chip is given by a combination of the cooling power from the coldfinger and the heating power due to thermal radiation from the environment. We find a very good agreement between the experimentally determined resonance frequencies shown in Supplementary Figure 4b, the transition temperature of our Nb $T_c = 9.2\,\mathrm{K}$ and Supplementary Eq. (9) when we assume $T_{\mathrm{off}} = 1.05\,\mathrm{K}$, $\omega_{\mathrm{Res0}} = 2\pi \cdot 6.94378\,\mathrm{GHz}$ and a kinetic inductance participation ratio $L_k(T=0)/L_0 = 0.02589$. The result is shown as black line in Supplementary Figure 4b and gives us a rough estimate for the temperature offset between sample and sensor.

B. Temperature fine calibration and full cavity characterization

As the offset temperature T_{off} is not exactly constant between 5 K and 9 K and as all our experiments are done within a limited temperature window of $\sim 1\,\mathrm{K}$, we performed a more detailed cavity characterization in the corresonding temperature interval. The results of this detailed cavity characterization are shown in Supplementary Figure 5. In Supplementary Figure 5a, we plot the resonance frequency vs the sample temperature, where the sample temperature was determined from the analytical approximation shown as black line. To achieve the best match in this temperature region, we had to adjust the offset temperature to $T_{\mathrm{off}} = 1.09\,\mathrm{K}$, but kept all other parameters used above.

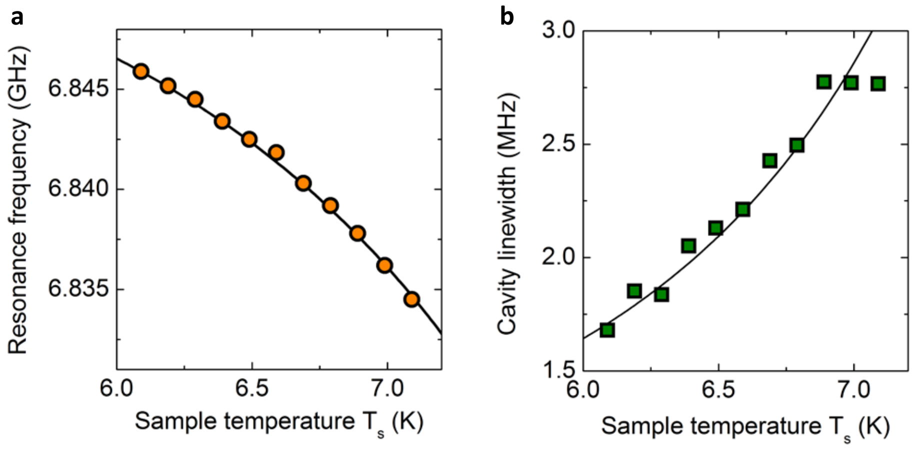

Supplementary Figure 5: **Temperature dependence of the cavity parameters**. **a** Cavity resonance frequency $\omega_{\mathrm{Res}}/2\pi$ vs sample temperature. Circles are data extracted from the measurements and the black line is an analytical approximation curve (details see text). **b** Cavity linewidth $\kappa/2\pi$ vs sample temperature extracted from Lorentzian fits. Squares are experimental data and the black line is an approximation based on the two-fluid model (details see text). The data point at 6.99 K is linearly interpolated from points at 6.89 K and 7.09 K.

In addition to the resonance frequency, we also extracted the resonance linewidth κ for each temperature, which is shown in Supplementary Figure 5b. From the two-fluid model [3, 5], it follows that the surface resistance of a superconductor is given by

$$R_s = \frac{1}{2}\omega^2 \mu_0^2 \sigma_1 \lambda_{\mathrm{L}}^3 \tag{11}$$

where $\sigma_1 \propto n_n/n_e$ is the real part of the complex two-fluid conductivity with the quasiparticle density n_n and the total electron density n_e. From the temperature dependence of λ_{L} and the two-fluid model, the temperature dependence of the superconducting charge carrier density is given by

$$\frac{n_s(T)}{n_e} = 1 - \left(\frac{T_s}{T_c}\right)^4 . \tag{12}$$

This leads to the quasiparticle density fraction

$$\frac{n_n(T)}{n_e} = \left(\frac{T_s}{T_c}\right)^4 . \tag{13}$$

Taking the relation $\kappa_s \propto R_s$ for the quasiparticle induced losses and assuming $\omega_{\text{Res}}, L_{\text{tot}} \approx \text{const.}$, which for this consideration is reasonable as their relative change is only $\sim 10^{-2}$, we get as cavity linewidth temperature dependence

$$\kappa(T) = \kappa_0 + \kappa_1 \left(\frac{T_s}{T_c}\right)^4 \cdot \left[1 - \left(\frac{T_s}{T_c}\right)^4\right]^{-\frac{3}{2}} \tag{14}$$

with a temperature independent contribution κ_0 and the scaling factor κ_1. Figure 5b shows an approximation to the data using this expression with $\kappa_0 = 2\pi \cdot 850\,\text{kHz}$ and $\kappa_1 = 2\pi \cdot 3.25\,\text{MHz}$ ($T_{\text{off}} = 1.09\,\text{K}$) as lines.

C. Influence of the magnetic trapping fields

Applying an external magnetic field can shift the cavity frequency as well as the cavity linewidth due to Meissner screening currents [6] and the presence of Abrikosov vortices [7, 8]. In our experiment, we apply only small fields in the $100\,\mu\text{T}$ range, but due to the fact that we also apply a field during the transition to the superconduting state, we will trap some vortices in the cavity leads [9]. As the magnetic field distribution including vortices is very complicated for our device, we describe the field-induced property shifts phenomenologically by slightly adjusting the kinetic inductance participation ratio L_k/L_0 and the parameter κ_1.

In Supplementary Figure 6a, we plot the zero magnetic field data points and the analytic expressions (lines) as derived in the previous section and in 6b we show the experimental data obtained within the full magnetic trapping field configuration. For comparison, we also plot the lines of 6a in 6b, but in grey, demonstrating that the magnetic fields indeed lead to a small resonance frequency downshift and a slight increase of the linewidth. Both effects can be captured by using $L_k(B_{\text{trap}})/L_0 = 0.02593$ and $\kappa_1(B) = 1.3\kappa_1(0)$. The result is shown as black dashed lines in 6b and is in excellent agreement with the data.

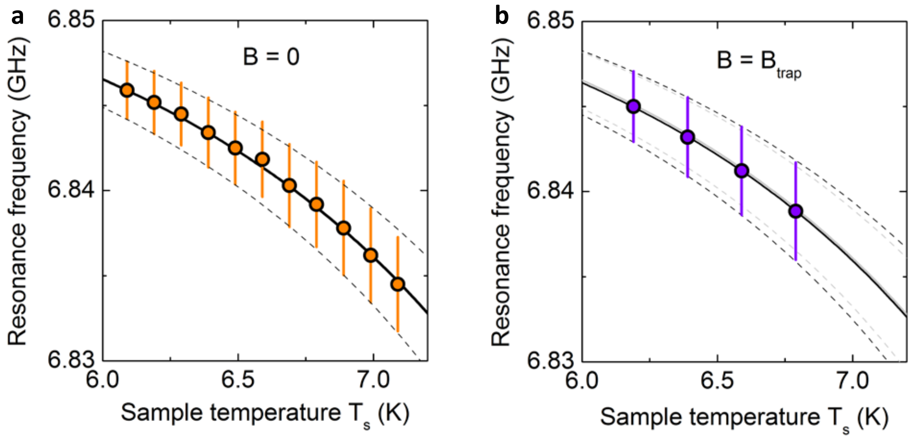

Supplementary Figure 6: **Temperature and magnetic field dependence of the cavity parameters in the experimentally relevant range**. **a** Cavity resonance frequency and linewidth vs sample temperature in zero magnetic field. Circles show the resonance frequency values, error bars on both sides of the points indicate the range $(\omega_{\text{Res}} \pm \kappa)/2\pi$. Lines show corresponding analytical approximations as described in the main text. **b** Cavity resonance frequency and linewidth vs sample temperature with magnetic trapping fields applied. Circles show the resonance frequency values, error bars on both sides of the points indicate the range $(\omega_{\text{cav}} \pm \kappa)/2\pi$. Gray lines show the corresponding analytical approximations for $B = 0$ as in a, and black lines indicate slightly modified expressions as described in the main text.

SUPPLEMENTARY NOTE 4. MAGNETIC FIELD SIMULATIONS

The magnetic field simulations in this work have been performed using the software package 3D-MLSI [2]. For the calculations of the RF magnetic field, simplified versions of our real chip were used, as the full structure was too large to be computed to the full extent. We do not expect the modifications (e.g. shortening the Z-shaped trapping wire to the trapping region), however, to have a significant impact onto the final results.

A. Coupling per photon and atom

The microwave current of the fundamental mode along the resonator is given by

$$I(l) = I_0 \cos\left(2\pi \frac{l}{\lambda_0}\right) \tag{15}$$

where l is the coordinate along the resonator starting from the input port with $l = 0$, $\lambda_0 \approx 18.7\,\mathrm{mm}$ is the resonance wavelength and I_0 is the amplitude in the current antinodes. To calculate the coupling rate g between a single photon and a single atom in the cavity, we estimate the zero point fluctuations of the microwave current in the resonator and at the position of the atoms (current antinode) by

$$\frac{1}{2}\hbar\omega_{\mathrm{cav}} = \int_0^{\lambda_0/2} L' I_{\mathrm{zpf}}^2 \cos^2\left(2\pi\frac{l}{\lambda_0}\right) dl \tag{16}$$

$$= \frac{\lambda_0}{4} L' I_{\mathrm{zpf}}^2 \tag{17}$$

where the inductance per unit length is $L' = 409\,\mathrm{nH/m}$ (kinetic inductance contributions are neglected here due to their smallness) and $I_{\mathrm{zpf}} = I_{\mathrm{zpf0}}/\sqrt{2}$ is the root mean square of the zero point fluctuation amplitude I_{zpf0}. With $\omega_{\mathrm{cav}} = 2\pi \cdot 6.84\,\mathrm{GHz}$ and $\lambda_0 \approx 18.7\,\mathrm{mm}$ we get

$$I_{\mathrm{zpf}} = \sqrt{\frac{2\hbar\omega_{\mathrm{cav}}}{\lambda_0 L'}} \approx 33.5\,\mathrm{nA}. \tag{18}$$

To relate this to the coupling, we calculate the magnetic field B_{ph} related to this current at the position of the atoms by means of finite element simulations using the software package 3D-MLSI [2].

Finally, we take into account the position of the atomic cloud along the resonator, which reduces the effective magnetic field to $\sim 0.95 B_{\mathrm{ph}}$. Supplementary Figure 7 shows the magnetic microwave field zero point fluctuations obtained from these simulations in a cross-section of the coplanar waveguide at the position of the atoms.

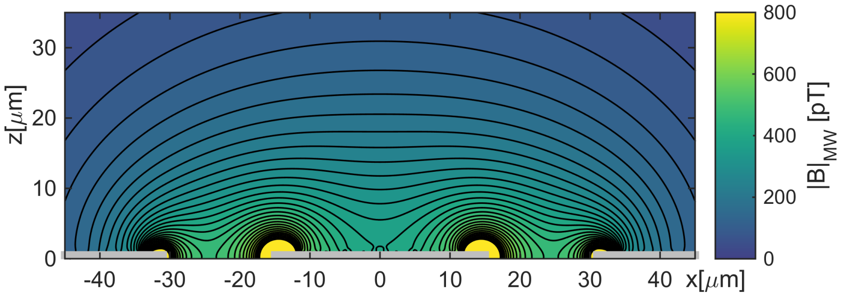

Supplementary Figure 7: **Single-photon microwave magnetic field in the resonator**. The magnetic microwave field zero point fluctuation amplitude $|B| = |B_{\mathrm{ph}}|$ [nT] obtained by finite element simulations above the coplanar microwave structure. The coplanar waveguide structure is indicated by the grey bars at the bottom. The thickness of the CPW is not to scale.

From the magnetic microwave field, we calculate the single-atom coupling rate as

$$g = \frac{|B_{\mathrm{ph}}(x, y)| \cdot |\mu|}{\hbar} \tag{19}$$

with the magnitude of the dipole transition matrix element $|\mu| = 0.25\mu_{\mathrm{B}}$. The result is shown in Fig. 1f of the main paper.

B. The radio-frequency magnetic field

For the two-photon experiments and the corresponding simulations, we also need the magnetic field of the radio-frequency (RF) current, which is sent through the Z-shaped trapping wire. Thus, we calculate the magnetic field for

a current of $I_{\mathrm{RF}} = 1\,\mathrm{mA}$ on the trapping wire and show the result at the position of the atoms in Supplementary Figure 8.

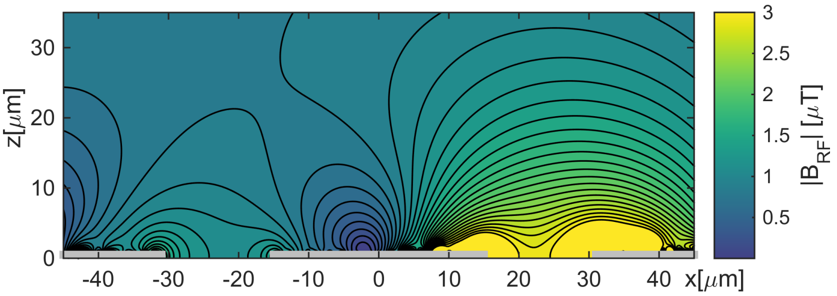

Supplementary Figure 8: **Magnetic field of a current through the trapping wire in the resonator**. The plot shows the magnetic field $|B_{\mathrm{RF}}|$ [μT] generated in the microwave cavity, when a current of $I_{\mathrm{RF}} = 1\,\mathrm{mA}$ is flowing through the trapping wire. The grey bars at the bottom indicate the position of the resonator leads. The centre bar shows the centre conductor, the left bar corresponds to the $50\,\mu$m ground plane towards the trapping wire and the right bar corresponds to the large purely superconducting ground. The magnetic field is mainly guided through the right resonator gap due to the closed superconducting loop around the left gap (defined by centre conductor, ground and the coupling inductors).

SUPPLEMENTARY NOTE 5. RECONSTRUCTION OF THE CAVITY FIELD WITH RAMSEY INTERFEROMETRY

In order to measure the cavity field strength as depicted in Fig. 2 in the main article, we prepare the atoms in a superposition of the states $|1,-1\rangle$ and $|2,1\rangle$ with a two-photon $\pi/2$-pulse using external radio- and MW frequencies ω_{RF} and ω_{extMW}. After a variable waiting time T_{Ramsey}, a second $\pi/2$-pulse is irradiated and the population in the two hyperfine states is measured. The two-photon detuning with respect to the atomic transition frequency is chosen to be $\omega_{\mathrm{Ramsey}} = \omega_{\mathrm{at}} - (\omega_{\mathrm{RF}} + \omega_{\mathrm{extMW}}) = -2\pi \cdot 500\,\mathrm{Hz}$. The presence of a microwave field in the cavity shifts the atomic transition frequency to $\omega_{\mathrm{at}} + \delta_{\mathrm{dress}}$, so the measured frequency in the Ramsey sequence changes. Supplementary Figure 9 shows two exemplary Ramsey measurements taken at a chip temperature of 6.4 K. The first

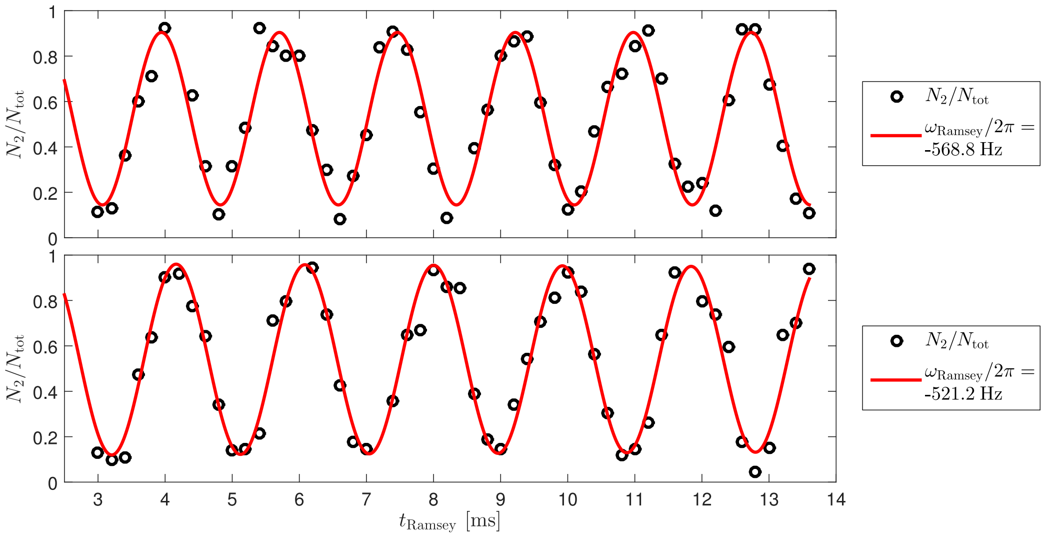

Supplementary Figure 9: **Recorded Ramsey fringes for different resonator driving frequencies.** Shown are two exemplary measurements for dressing frequencies, both recorded at a chip temperature of 6.4 K. Top: $\omega_{\mathrm{dress}} = 2\pi \cdot 6.838\,\mathrm{GHz}$ yielding a shift of $\delta_{\mathrm{dress}}/2\pi = -68.8\,\mathrm{Hz}$. Bottom: $\omega_{\mathrm{dress}} = 2\pi \cdot 6.843\,\mathrm{GHz}$, yielding a shift of $\delta_{\mathrm{dress}}/2\pi = -21.2\,\mathrm{Hz}$.

measurement yields a shift of $\delta_{\mathrm{dress}}/2\pi = -68.8\,\mathrm{Hz}$ for a driving field of $\omega_{\mathrm{dress}} = 2\pi \cdot 6.838\,\mathrm{GHz}$, the second one yields $\delta_{\mathrm{dress}}/2\pi = -21.2\,\mathrm{Hz}$ for $\omega_{\mathrm{dress}} = 2\pi \cdot 6.843\,\mathrm{GHz}$. b7b1c3ff

SUPPLEMENTARY NOTE 6. SIMULATED RABI OSCILLATIONS IN THE CAVITY

A. One photon Rabi oscillations

Numerical simulations of the coherent Rabi oscillations of atomic ensembles in the cavity yield further insight into the observed dephasing rates. We assume a thermal ensemble of atoms with a temperature of $T_a = 2000\,\mathrm{nK}$ trapped in a harmonic magnetic trap with $\omega_x = 2\pi \cdot 400\,\mathrm{s}^{-1}$, $\omega_y = 2\pi \cdot 25\,\mathrm{s}^{-1}$, $\omega_z = 2\pi \cdot 600\,\mathrm{s}^{-1}$. The centre of the trap is assumed $20\,\mu$m from the chip surface, as depicted in Fig. 1f in the main article.

For the one-photon Rabi oscillation, the Rabi frequency is much higher than the oscillation frequency of the atoms in the trapping potential, i.e. $\Omega_0 \gg \omega_z$. We therefore can assume a static Gaussian density distribution of atoms in the trap, and use a total atom number of 1.2×10^5 atoms for the simulations. We use the numerically calculated field strength depicted in Supplementary Figure 7, multiplied by a constant numerical factor to match the observed Rabi oscillation frequency. For each position $\mathbf{r}_i$, the probability to find atoms in the excited state is computed as

$$p_2(\mathbf{r}_i, t) = \frac{\Omega_0(\mathbf{r}_i)^2}{\tilde{\Omega}(\mathbf{r}_i)^2} \sin^2\left(\frac{\tilde{\Omega}(\mathbf{r}_i)^2}{2} t\right), \tag{20}$$

where $\tilde{\Omega}(\mathbf{r}_i)^2 = \Omega_0(\mathbf{r}_i)^2 + \Delta(\mathbf{r}_i)$ is the generalized Rabi frequency, and $\Delta(\mathbf{r}_i)$ the magnetic-field dependent detuning of the microwave to the atomic transition. The probability $p_2(\mathbf{r}_i, t)$ is multiplied with the local atomic density $n_{\mathrm{at}}(\mathbf{r}_i)$ and summation over all atoms yields the total atom number in the excited state. The simulated results closely match the observed dephasing of the Rabi oscillations, as seen in Fig. 3a in the main paper.

B. Two-photon Rabi oscillations

For the simulated two-photon Rabi oscillations, we assume a three level system of states $|1,-1\rangle$, $|2,0\rangle$, and $|2,1\rangle$. States $|1,-1\rangle$ and $|2,0\rangle$ are coupled by the cavity microwave field with the Rabi frequency Ω_{MW}. An additional radio frequency Ω_{RF} couples the state $|2,0\rangle$ to the state $|2,1\rangle$. Both the microwave and the radio frequency field are detuned to the transition to the intermediate state $|2,0\rangle$ by the detuning $\pm\Delta$, c.f. Fig. 5b in the main article. The inhomogeneity of the cavity field Ω_{MW} is the same as for the one-photon case above. The spatial dependence of the radio-frequency field Ω_{RF} is simulated with the software package 3D-MLSI by applying a current in the Z-shaped wire and calculating the Meissner screening currents close to the resonator, c.f. 8. As the effective Rabi frequency is much lower as in the one-photon case, the assumption of static atoms no longer holds. The motion of atoms through the spatially inhomogeneous MW and RF field leads to a time dependence of the Rabi frequency seen by each atom.

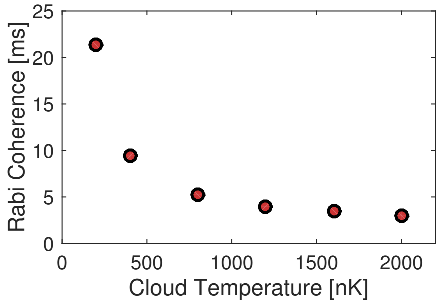

Supplementary Figure 10: **Simulated coherence of the Rabi oscillations as a function of the cloud temperature.** The simulations assume trapping frequencies as in the experiment, i.e. $\omega_y = 2\pi \cdot 25\,\mathrm{s}^{-1}$, $\omega_x = 2\pi \cdot 400\,\mathrm{s}^{-1}$, $\omega_z = 2\pi \cdot 600\,\mathrm{s}^{-1}$. The high coherence for low temperatures shows that the inhomogeneity of the MW field is the primary source of the dephasing.

.

To account for this, we randomly initialize 5000 non-interacting particles in the state $|1,-1\rangle$ in the harmonic potential with a distribution corresponding to a temperature of 800 nK. We then simulate the movement of the atoms through the potential and the evolution of the three states with a Runge-Kutta calculation of fourth order. Stability of the simulations was ensured by changing the time steps in the calculations. The main source of the dephasing in the Rabi oscillations is the inhomogeneity of the MW field. This can be seen from simulations with colder and thus

smaller clouds, as visible in Supplementary Figure 10.

SUPPLEMENTARY REFERENCES

[1] Bothner, D. et al., Inductively coupled superconducting half wavelength resonators as persistent current traps for ultracold atoms, *New J. Phys.* **15**, 093024 (2013)

[2] Khapaev, M. M., Kupriyanov, M. Y., Goldobin, E., and Siegel, M., Current distribution simulation for superconducting multi-layered structures, *Supercond. Sci. Technol.* **16**, 24 (2003)

[3] Tinkham, M. *Introduction to Superconductivity: Second Edition*, Dover Books on Physics (Dover Publications, 2004)

[4] Cano, D. et al., Experimental system for research on ultracold atomic gases near superconducting microstructures, *Eur. Phys. J. D* **63**, 17-23 (2010)

[5] Buckel, W. and Kleiner,R. *Superconductivity: fundamentals and applications* (Wiley-VCH, 2004)

[6] Healey, J. E., Lindström, T., Colclough, M. S., Muirhead, C. M., and Tzalenchuk, A. Ya., Magnetic field tuning of coplanar microwave resonators, *Appl. Phys. Lett.* **93**, 043513 (2008)

[7] Song, C. et al., Microwave response of vortices in superconducting thin films of Re and Al, *Phys. Rev. B* **79**, 174512 (2009)

[8] Bothner, D. et al., Magnetic hysteresis effects in superconducting coplanar microwave resonators, *Phys. Rev. B* **86**, 014517 (2012)

[9] Stan, G., Field, S. B., and Martinis, J. M., Critical field for complete vortex expulsion from narrow superconducting strips, *Phys. Rev. Lett.* **92**, 097003 (2004)
